# Factors Influencing Walking Improvement After Immersive Virtual Reality Training with a Bodyweight-Supporting System in Poststroke Patients: A Prospective Single-Arm Study

**DOI:** 10.3390/neurolint18090162

**Published:** 2026-08-24

**Authors:** Kanta Kitabayashi, Naoto Ogura, Naoki Yoshioka, Wataru Kakuda

**Affiliations:** 1 Department of Rehabilitation, International University of Health and Welfare Ichikawa Medical Center, Chiba 272-0827, Japan; kanta@ihwg.jp (K.K.); naoto10134568@ihwg.jp (N.O.); 2 Department of Rehabilitation Medicine, Graduate School of Medicine, International University of Health and Welfare, Chiba 286-8686, Japan; 3 Department of Radiology, International University of Health and Welfare Narita Hospital, Chiba 286-8520, Japan; nyoshioka@ihwg.jp; 4 Department of Rehabilitation Medicine, International University of Health and Welfare School of Medicine, Chiba 286-8686, Japan

**Keywords:** virtual reality (VR), balance function, rehabilitation, walking function, stroke

## Abstract

**Objectives:** We developed an original protocol for standing balance and walking function training using immersive virtual reality (IVR) combined with a bodyweight-supporting (BWS) system. The purpose of this study was to evaluate the feasibility of IVR training in poststroke patients and to identify clinical characteristics associated with walking improvement. **Methods**: A total of 22 poststroke patients admitted to a rehabilitation ward were enrolled. All participants received conventional physical therapy daily, and IVR training was initiated when balance recovery plateaued. The original IVR training was conducted for 10 consecutive days using a BWS system, enabling safe and repetitive practice of standing weight shifting and advanced stepping tasks, with task difficulty individually adjusted. Primary outcomes were the Berg Balance Scale (BBS), Functional Reaching Test (FRT), and walking time on the 10-m walk test (10 MWT). Changes in 10 MWT (Δ10 MWT) were calculated, and participants were dichotomized into good and poor walking improvement groups based on Δ10 MWT to compare baseline clinical characteristics (e.g., BBS, FRT, and Functional Ambulation Categories [FAC]) between the groups. Receiver operating characteristic (ROC) curve analyses were conducted to identify cutoff values associated with greater improvements in walking. **Results**: BBS, FRT, and 10 MWT improved significantly after the 10-day IVR training (*p* < 0.01). ROC analyses identified FAC ≤ 3 and BBS ≤ 42 before training as cutoff values associated with greater improvement in walking performance. **Conclusions**: Our proposed IVR training approach using a BWS system could be safely implemented and might improve standing balance and walking function in poststroke patients with a balance disability.

## 1. Introduction

Stroke is one of the major causes of physical disabilities, and approximately 80% of poststroke patients suffer from disabilities relating to motor, language, cognitive, behavioral, and swallowing function [1,2]. Impairments in standing balance and walking function are very often found, and approximately 70% of poststroke patients living at home experience a fall within 1 year after the onset of a stroke [3,4]. Consequently, experiencing a fall can lead to a decrease in the quality of life (QOL), since some patients have a fear of falling and limit their activities [5,6]. Therefore, rehabilitative management for standing balance and walking function is quite important for improving QOL among poststroke patients.

Recently, virtual reality (VR) training has emerged as one of the novel therapeutic tools for treating stroke, Parkinson’s disease, and spinal cord injuries, not only in Japan but also around the world [7,8,9,10]. However, most studies have focused on non-immersive VR systems, such as computer displays and commercial games [8,11]. Recent systematic reviews have demonstrated the potential of immersive VR (IVR) for upper-limb rehabilitation [12] and rehabilitation for unilateral spatial neglect after stroke [13]. However, a recent systematic review of IVR interventions in poststroke rehabilitation included relatively few studies on standing balance and walking rehabilitation [14]. Accordingly, it seems uncommon to perform IVR training in the standing position to train standing balance and walking function because of concerns regarding safety and the risk of falls. So far, the combined use of a bodyweight-supporting (BWS) system with IVR training for standing balance and walking rehabilitation has not been reported. Consequently, we developed an original protocol for standing balance and walking function training using IVR in combination with a BWS system. The combined use of IVR and BWS is expected to enable safe engagement for challenging tasks in the standing position.

Furthermore, it remains unclear which baseline clinical characteristics are associated with greater walking improvement following IVR training. It is important to identify which patients are most likely to benefit from IVR training in order to optimize patient selection. The purpose of this study was to evaluate the safety and efficacy of our proposed IVR training with a BWS system and to identify baseline clinical characteristics associated with greater walking improvement following IVR training.

## 2. Materials and Methods

### 2.1. Participants

This study was a single-center, prospective, single-arm interventional study. Between February 2024 and November 2025, 22 consecutive poststroke patients admitted to the rehabilitation ward at International University of Health and Welfare Ichikawa Medical Center who met the inclusion criteria were enrolled in this study. All participants had been admitted for long-term inpatient rehabilitation. IVR training was conducted in the training room of our rehabilitation ward.

The inclusion criteria for this study were as follows: (1) stroke in subacute to chronic phase following completion of acute stroke management, (2) clinical diagnosis of ischemic or hemorrhagic stroke presenting with mild-to-moderate hemiparesis or balance disability at the time of admission to our ward, (3) age between 20 and 90 years at admission, (4) Brunnstrom Recovery Stage 4–6 for lower limbs at the time of study inclusion, (5) being able to maintain sitting and standing positions for a couple of minutes, (6) being able to understand the purpose and protocol of this study, and (7) being able to obtain written consent. The exclusion criteria were as follows: (1) severe cognitive and visual impairment, (2) past history of seizure, and (3) orthopedic or medical diseases that can significantly limit physical activity.

The protocol of our IVR training and some evaluations conducted in this study were approved by the Ethics Committee of International University of Health and Welfare (approval number: 23-Im-017) and registered with the University Hospital Medical Information Network (UMIN) center (UMIN-CTR ID: R000060821 UMIN000053291). This study was conducted in compliance with the Declaration of Helsinki, and written informed consent was obtained from all participants before study inclusion.

### 2.2. Intervention

After admission to our ward, first, all participants received 60 min of conventional physical therapy daily. During this period, the Berg Balance Scale (BBS) was evaluated weekly. After balance recovery reached a plateau state, defined as no increase in the BBS score during the preceding week, IVR training was initiated in addition to conventional physical therapy. Each IVR session was scheduled for approximately 20 min, including preparation, equipment setup, and a break between sets. The actual IVR training consisted of two 5-min training sets (10 min in total), followed by 40 min of conventional physical therapy. IVR training was provided once daily for 10 consecutive days (10 sessions in total). An approximately 3-min break was scheduled between the two training sets, during which blood pressure, heart rate, and other vital signs were assessed as needed. Throughout their hospitalization, all participants were monitored continuously, with special attention to adverse events associated with IVR training such as dizziness, nausea, and seizure attacks.

**Figure 1:**
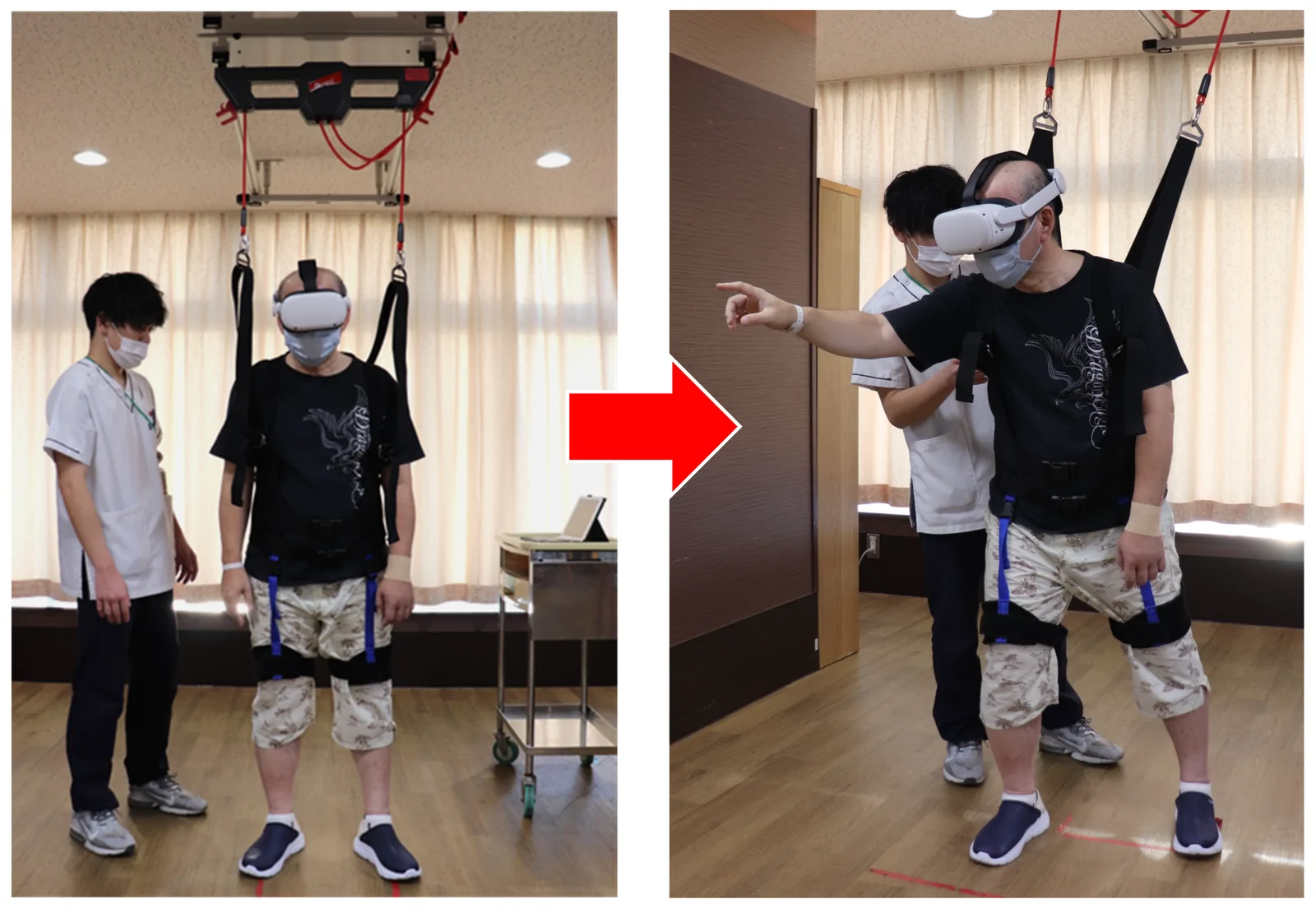
Immersive virtual reality training with a bodyweight-supporting system. Participants were fitted with a bodyweight-supporting system to prevent falls during the training and were carefully monitored by a trained physical therapist.

For this study, we used Meta Quest 2 (Meta Platforms, Menlo Park, CA, USA), with IVR images being projected onto an iPad (Apple, Cupertino, CA, USA), which allowed the therapist to monitor the virtual space simultaneously during the training shown in Figure 1. This IVR training was conducted without holding a controller. During their training, importantly, all participants were equipped with a BWS system to ensure safe performance of high-difficulty balance tasks in the standing position and prevention of falls during the training. The BWS system used in this study was a ceiling-mounted device comprising an adjustable rope and a trunk harness. Before each session, a physical therapist fitted the participants with the harness, which was securely fastened around the participant’s trunk and hips. The rope length was adjusted according to each participant’s height, allowing for natural posture and movement. If needed, the physical therapist also fitted the participants with a short leg brace.

**Figure 2:**
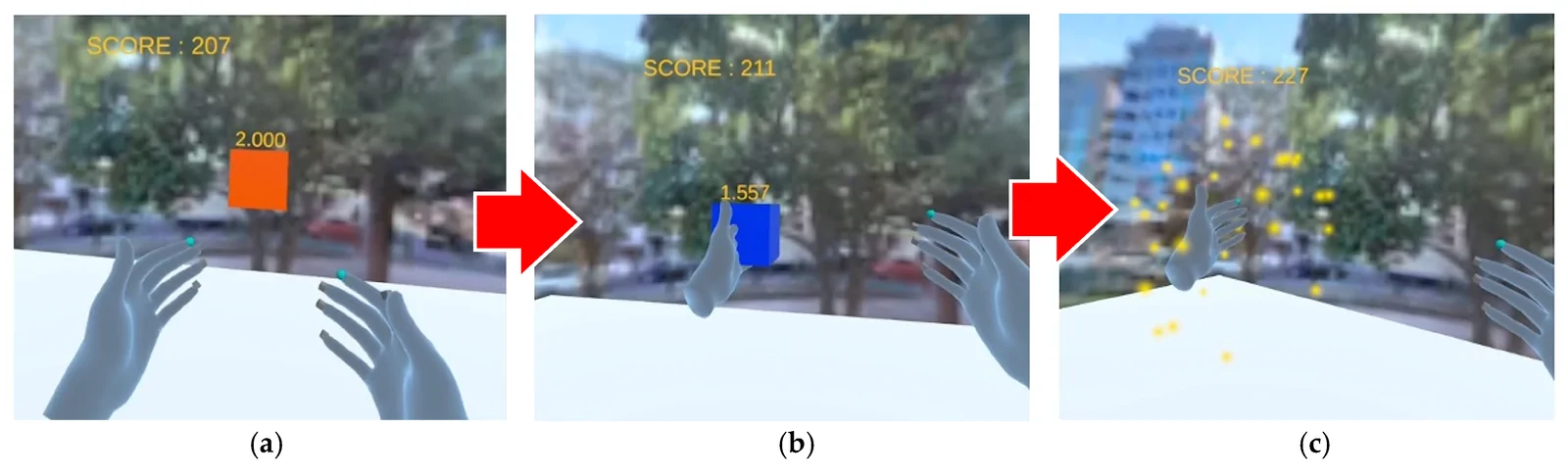
A typical image of the virtual environment during training. The participant’s hands and orange targets are projected on the virtual image (**a**). When the participant successfully reaches and touches the target, the color of the target changes (**b**). Finally, if the target is touched for 2 s, a small yellow explosion appears, followed by a distinct sound produced by the Meta Quest 2 speaker (**c**).

During the IVR training, both the participant’s hands and orange box targets were presented on the IVR displays as shown in Figure 2a. Thereafter, participants were instructed to touch static and dynamic box-shaped targets which appeared vertically and horizontally, with their left and right hands. This IVR training method was proposed to facilitate center-of-gravity shift and step movements in the standing position through the participant’s attempt to touch the target. As feedback to the participant, the color of the target changed when successfully touched shown in Figure 2b,c, followed by a distinct sound. Moreover, the time that the participants were able to touch the 1 set was displayed as the VR score. The IVR score is added 1 point for every 0.1 s when the target is successfully touched by the participant’s hand.

For IVR training, 3 difficulty levels, namely, “easy mode”, “normal mode”, and “hard mode”, were prepared. The difficulty of the task was lowest in easy mode and highest in hard mode. At first, easy mode was applied for all participants. The task progressively became more difficult as the VR score improved, specifically when a participant scored at least 1000 points for 1 set on 2 consecutive days. As the difficulty increased, reaching distance extended, and the targets appeared on the image more frequently, moving faster. In hard mode, the targets sometimes rotated horizontally, requiring participants to twist and step backward. This IVR application was uniquely developed in Unity2021.3.1f1 (Unity Technologies, San Francisco, CA, USA) with study collaborators.

### 2.3. Outcome Measures

As primary outcomes, 3 objective assessment scales, namely, BBS, Functional Reaching Test (FRT), and walking time on the 10-m walk test (10 MWT), were applied. These were evaluated by a physical therapist who was blinded to the participants’ information before the first IVR training session and after the 10 sessions. The BBS, which comprehensively assesses static and dynamic balance function, has a maximum total score of 56, with a score of <45 having been reported to be associated with a high risk of falling [15,16]. The FRT measures the distance (cm) of a maximal forward reach in the standing position [17]. Participants must stretch their arms forward as far as possible while maintaining their base of support, and longer distances indicate better balance function [17]. The 10 MWT measures the walking time (seconds) at the fastest possible safe speed [18]. Participants were evaluated before and after IVR training under similar measurement conditions (if needed, walking aids and lower-limb braces were applied) to ensure reliability.

As secondary outcomes, we also evaluated 2 objective assessment and 3 self-assessment scales. The Brunnstrom Recovery Stage (BRS) was used to assess motor recovery of the lower limb, ranging from stage I (flaccidity) to stage VI (near-normal movement) [19,20]. The Functional Ambulation Categories (FAC) was used to evaluate walking function, with scores ranging from 0 (non-functional ambulation) to 5 (independent ambulation) [21]. These were evaluated by a physical therapist before the first IVR training session and after the 10 sessions. The Mini-Mental State Examination (MMSE) was used to assess global cognitive function at admission. Scores range from 0 to 30, with higher scores indicating better cognitive function [22].

The Simulator Sickness Questionnaire (SSQ) was used to evaluate the adverse effects associated with the proposed training based on 16 symptoms (i.e., headache, nausea, and eyestrain) on a 4-point Likert scale [23]. Accordingly, a higher SSQ score indicates more severe adverse effects [23]. The visual analogue scale (VAS) was applied to evaluate the participants’ satisfaction, enjoyment, and immersion with our proposed training. The VAS scores were determined using a 10 cm horizontal line with a “score of 0” (i.e., “no satisfaction, enjoyment, and immersion”) written on the left end, whereas a “score of 100” (i.e., “highest satisfaction, enjoyment, and immersion”) written on the other end [24,25]. The SSQ and VAS scores were evaluated after completing all 10-day IVR training sessions. Furthermore, the Fall Efficacy Scale (FES) was applied to evaluate the fear of falling by determining the participants’ confidence in performing a 10-item activity without falling. The scores were graded from 10 to 40, with lower scores indicating a greater fear of falling [26]. The FES was evaluated before the first IVR training session and after completing all 10 sessions.

### 2.4. Statistical Analysis

Continuous variables were expressed as mean ± standard deviation (SD) or median and interquartile range (IQR), as appropriate. Age, time from onset to admission, time from admission to IVR training, SSQ score, and VAS scores were expressed as mean ± SD, whereas other clinical variables, including Functional Independent Measure (FIM), MMSE, BBS, FRT, BRS, FAC, and FES were expressed as median (IQR). Changes in the BBS score, FRT, 10 MWT, BRS, FAC, and FES score before and after the 10-day IVR training were analyzed using the Wilcoxon signed-rank test.

Participants were dichotomized into good and poor improvement groups based on the median value of the change in 10 MWT (Δ10 MWT). The Δ10 MWT was calculated as post-training minus pre-training values; thus, larger negative values indicated greater improvement. The median Δ10 MWT was −3.15 s; participants with a Δ10 MWT ≤ −3.15 s were classified as the good improvement group, whereas those with a Δ10 MWT > −3.15 s were classified as the poor improvement group. Between-group comparisons were performed using the Mann–Whitney U test. Fisher’s exact test was used for categorical variables (gender, type of stroke, site of stroke lesion, side of stroke lesion).

Receiver operating characteristic (ROC) curve analysis was performed to evaluate the ability of baseline variables to discriminate between good and poor walking improvement groups. The area under the curve (AUC), 95% confidence intervals (CI), sensitivity, and specificity were calculated. The Youden index (sensitivity + specificity − 1) was used to determine optimal cutoff values.

All statistical analyses were performed using Statistical Package for Social Science version 23.0 (SPSS Inc., Chicago, IL, USA). A *p* value < 0.05 was considered statistically significant.

## 3. Results

Table 1 summarizes the basic characteristics of the participants. As described in the Methods Section, the difficulty level of IVR training was progressively adjusted based on performance. During the final session, hard mode was applied for 9 participants (9/22, 40.9%) due to improvements in task performance.

### 3.1. Primary Outcome Measures

**Figure 3:**
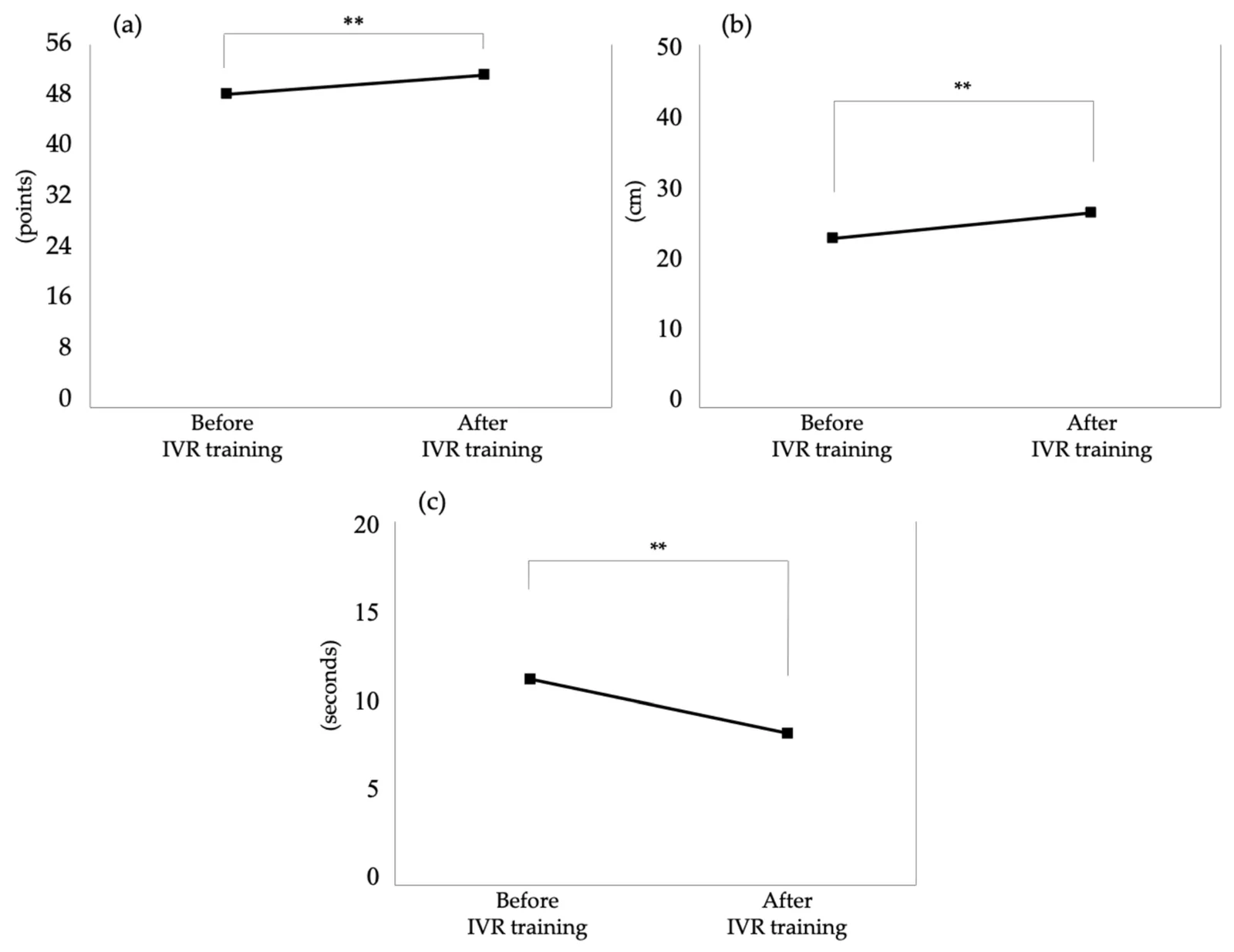
Changes in physical function with the proposed immersive virtual reality training protocol. Total BBS score (**a**), length in FRT (**b**), and walking time on the 10-m walk test (**c**). Data are presented as median values. Statistical analyses were performed using the Wilcoxon signed-rank test. The significance level was set at *p* < 0.05. ** A significant difference between conditions at *p* < 0.01.

Figure 3 shows the changes in the 3 primary outcome measures with our IVR training. Accordingly, the changes in all 3 parameters reflected the beneficial effects of our proposed IVR training protocol. The BBS score increased significantly from 48 (41–50) points to 51 (47–53) points after 10 sessions of the training, as shown in Figure 3a (*p* < 0.001). Moreover, our IVR training significantly increased the reach distance during the FRT from 23.0 (20.1–29.8) cm to 26.6 (24.0–32.9) cm, as shown in Figure 3b (*p* = 0.001). Finally, our training significantly shortened the 10 MWT time from 11.3 (8.1–20.2) to 8.2 (6.4–11.9) seconds, as shown in Figure 3c (*p* < 0.001). Among the included participants, 15 exhibited a reduction of more than 30% in 10 MWT time compared to that before the 10-day IVR training.

### 3.2. Secondary Outcome Measures

No significant change was observed in the BRS after the intervention (*p* = 0.32). In contrast, FAC showed significant improvement following the IVR training (*p* < 0.01). Regarding adverse effects, the average score for the total SSQ was 5.1 ± 5.7. The most common adverse effect was “fatigue,” which was reported by 11 participants (11/22, 50.0%). Other adverse effects experienced by the participants included “eyestrain” (3/22, 13.6%), “difficulty focusing” (3/22, 13.6%), “blurred vision” (3/22, 13.6%), “difficulty concentrating” (2/22, 9.1%), “general discomfort” (1/22, 4.5%), “increased salivation” (1/22, 4.5%), “nausea” (1/22, 4.5%), and “fullness of head” (1/22, 4.5%). However, all of these effects were mild and transient and did not need any medical care. Moreover, no falls were reported during the IVR training in this study. VAS scores for satisfaction, enjoyment, and immersion for our protocol were 81.3 ± 20.5, 81.8 ± 25.3, and 80.0 ± 23.3, respectively, which suggested that our training protocol promoted favorable levels of satisfaction, enjoyment, and immersion. Finally, our training significantly increased the FES score from 31 (26–36) to 35 (31–40) points (*p* < 0.001), indicating that the training decreased participants’ fear of falling.

### 3.3. Comparison Between Good and Poor Improvement Groups

Participants were dichotomized into good and poor walking improvement groups based on the median Δ10 MWT value (median: −3.15 s). The good improvement group demonstrated significantly lower baseline BBS (*p* = 0.028) and FAC scores (*p* = 0.007) compared with the poor improvement group, whereas no significant differences were observed in FIM, MMSE, FRT, BRS, and FES (*p* > 0.05) (Table 2).

### 3.4. ROC Curve Analysis

**Figure 4:**
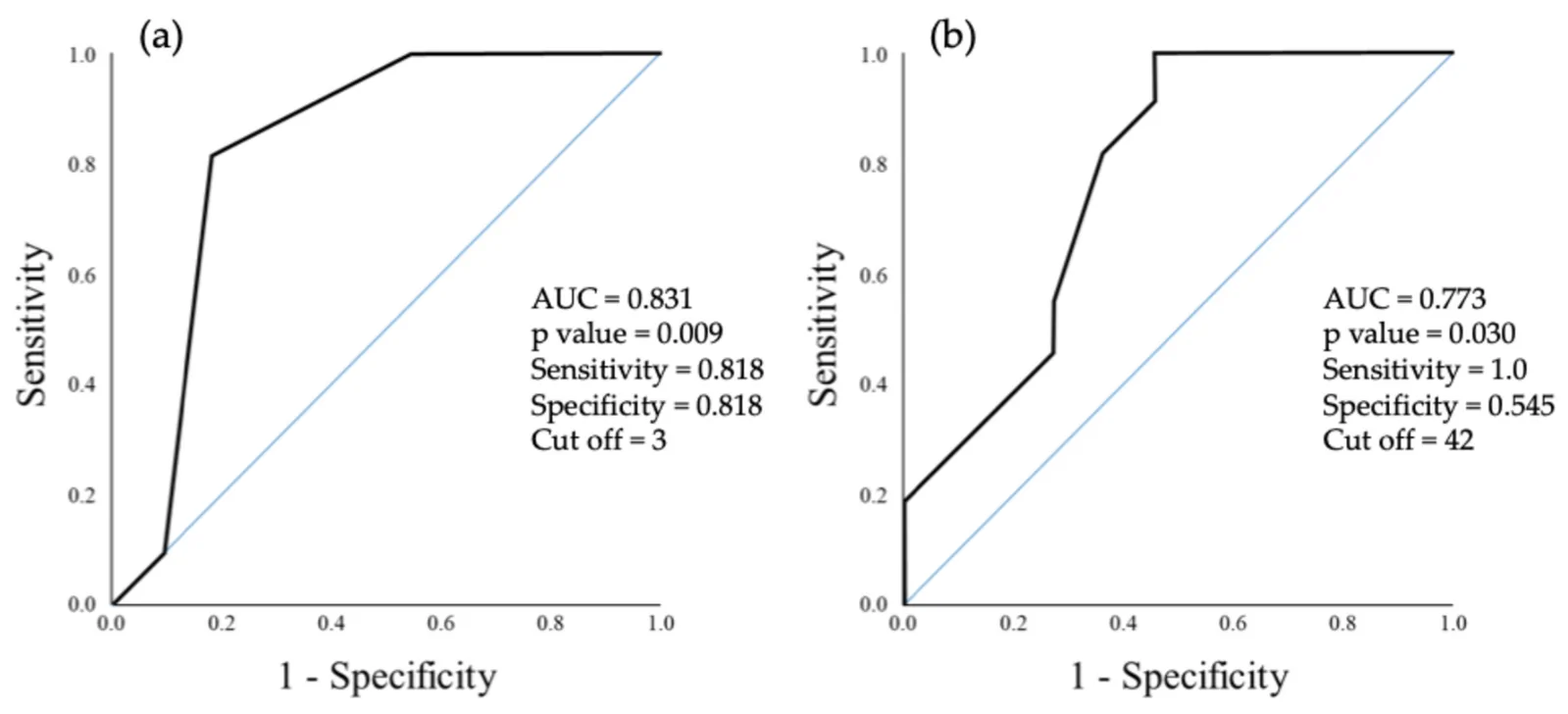
ROC analysis of FAC and BBS. Plots show cutoff values for: (**a**) FAC and (**b**) BBS for discriminating between good and poor improvement groups based on Δ10 MWT.

The results of ROC analysis are presented in Figure 4. For FAC, AUC was 0.83 (95% CI: 0.64–1.00), with an optimal cutoff value of ≤3 yielding a sensitivity of 81.8% and specificity of 81.8% (Figure 4a). For BBS, the AUC was 0.77 (95% CI: 0.57–0.98). A cutoff value of ≤42 points provided a sensitivity of 100% and specificity of 54.5% (Figure 4b). 

## 4. Discussion

The current study demonstrated acceptable safety, feasibility, and efficacy of IVR training with a BWS system for improving standing balance and walking function among poststroke patients. To our knowledge, this is the first study to report the clinical applicability of IVR training with a BWS system in this population.

The absence of serious adverse events and the low SSQ score indicate that our protocol was well tolerated. The mean total SSQ score in this study was 5.1 ± 5.7, which is substantially lower than previously reported values for IVR using HMDs (14.3–35.3) [27]. The use of the BWS system may have contributed to this favorable safety profile by allowing participants to perform challenging tasks without the risk of falls. In addition, high VAS scores for satisfaction, enjoyment, and immersion indicate good acceptability of the intervention. A key characteristic of IVR is the sense of presence, in which individuals experience the virtual environment as if it were real [28]. In this study, the use of an HMD and real-time hand tracking without controllers likely enhanced this immersive experience. Furthermore, participants received immediate multisensory feedback on their movements and could monitor their performance through VR-based scores. These features may have promoted sustained engagement and motivation by facilitating goal setting and repeated task practice. Previous studies have also shown that such feedback and score presentation enhance motivation and engagement during VR-based rehabilitation [29,30]. Taken together, these findings suggest that IVR training with a BWS system is both safe and well accepted by patients.

The present study demonstrated significant improvements in standing balance, as evidenced by the BBS and FRT. Task-oriented, repetitive practice is a key principle for promoting motor recovery after stroke [31,32]. In this study, participants repeatedly performed goal-directed reaching and stepping tasks in a standing position, with task difficulty individually adjusted across three levels. In addition, real-time multisensory feedback on movement accuracy and performance may have facilitated error-based motor learning and reinforced appropriate movement patterns. The use of a BWS system allowed participants to perform challenging tasks safely without fear of falling, thereby enabling a higher intensity of task-specific practice. This combination of safe task execution and feedback-driven repetition likely contributed to improvements in balance function. Furthermore, IVR-based feedback may have supported the refinement of planned movements and their consolidation through repeated practice, complementing conventional physical therapy.

In terms of walking function, a significant improvement in 10 MWT performance was observed. Previous studies have reported that balance function is closely associated with walking performance after stroke [33]. In this study, repeated practice of multidirectional weight shifting and stepping tasks likely enhanced dynamic balance control, which in turn contributed to improvements in walking function.

Goh et al. reported that poststroke patients are more likely to have a fear of falling than healthy individuals [34]. Furthermore, Kovács et al. suggested that a high fear of falling may reduce independence and increase anxiety and depressive symptoms [35]. Thus, fear of falling can hinder both physical and psychological recovery in poststroke patients. In the present study, the FES score significantly improved following IVR training, indicating a reduction in fear of falling. One possible explanation is that participants were able to recognize their progress through VR-based performance scores, which may have provided repeated successful experiences. Such experiences may have enhanced confidence in activities of daily living and contributed to a reduction in fear of falling. These findings suggest that IVR training may help address psychological barriers to mobility, in addition to improving physical function.

These findings suggest that lower baseline walking and balance function may predict greater improvement following IVR training. In the present study, cutoff values of FAC ≤ 3 and BBS ≤ 42 were identified, indicating that patients with limited walking independence and impaired balance function tended to achieve greater gains. Importantly, all participants were able to maintain unsupported standing for a couple of minutes, suggesting that the observed benefits were most pronounced in patients with preserved static standing ability but impaired dynamic balance and a high risk of falls. This finding may be attributed to the use of a BWS system enabled patients with impaired balance function, who are at high risk of falls, to safely perform dynamic standing tasks such as weight shifting and stepping. These patients may otherwise have limited opportunities to engage in sufficient task-specific training in the standing position. By reducing fall risk, the BWS system likely allowed a higher intensity and repetition of task-specific training, which are key factors in poststroke motor recovery [31,32]. In addition, patients with lower baseline balance function may have benefited more from IVR-specific features, such as real-time feedback, which facilitates error-based motor learning. In contrast, patients with relatively preserved balance function may have experienced a ceiling effect within this training protocol. The use of a harness may have restricted movement and reduced task difficulty, potentially limiting further improvements. For these patients, more challenging conditions, such as unstable surfaces or more complex tasks, may be required to optimize training effects.

This study has certain limitations. First, this was a small number of patients, and no control group was prepared. Therefore, the observed improvements may have been influenced by spontaneous recovery, particularly in patients with relatively mild motor impairment after stroke. Comparative studies such as randomized controlled design studies that include a large number of patients, are needed to confirm the efficacy of our proposed IVR training in poststroke patients. Second, the long-term effects were not evaluated in this study. Accordingly, we desire to clarify whether the beneficial effects could be maintained after the cessation of the 10-day IVR training. Third, we did not apply any neuroimaging investigations, such as functional magnetic resonance imaging and near-infrared spectroscopy. It would be very crucial to clarify the underlying mechanisms by which our proposed IVR training facilitated functional recovery of poststroke hemiparetic patients.

## 5. Conclusions

This exploratory study suggests that our proposed IVR training protocol with a BWS system is feasible and may contribute to improvements in standing balance and walking function in poststroke patients. In addition, lower baseline balance and walking function, as indicated by a BBS ≤ 42 and a FAC score ≤ 3, may be associated with greater improvements following IVR training. Further large-scale studies are needed to confirm these preliminary findings and to determine the optimal duration and frequency of the training for poststroke patients.

## Figures and Tables

**Table 1:** Basic information on the participants.

	All (*n* = 22)
Gender (F/M)	8/14
Age at admission (years)	69.0 ± 11.8
Type of stroke (ICH/CI)	8/14
Site of stroke lesion (SUP/SUB)	12/10
Side of stroke lesion (Right/Left)	9/13
Time from onset to admission (days)	38.4 ± 29.1
Time from admission to IVR training (days)	42.3 ± 26.1
FIM before IVR training (points)	98.0 ± 20.9
MMSE at admission (points)	28.3 ± 2.6

Data are presented as mean ± standard deviation. Abbreviations: ICH: intracerebral hemorrhage; CI: Cerebral Infarction; SUP: supratentorial; SUB: subtentorial; FIM: Functional Independence Measure; MMSE: Mini-Mental State Examination.

**Table 2:** Clinical characteristics of the participants.

	Good Improvement Group (*n* = 11)	Poor Improvement Group (*n* = 11)	*p* Value
Gender (F/M)	4/7	4/7	0.67
Age at admission (years)	67.0 ± 10.4	71.1 ± 12.3	0.48
Type of stroke (ICH/CI)	5/6	3/8	0.33
Site of stroke lesion (SUP/SUB)	5/6	7/4	0.39
Side of stroke lesion (Right/Left)	4/7	5/6	0.50
Time from onset to admission (days)	48.5 ± 35.0	28.2 ± 13.5	0.19
Time from admission to IVR training (days)	48.5 ± 24.3	36.1 ± 25.2	0.13
FIM before IVR training (points)	96 (74–104)	104 (98–120)	0.12
MMSE at admission (points)	30 (29–30)	29 (27–30)	0.52
BBS before IVR training (points)	39 (34–49)	49 (48–51)	0.028 *
FRT before IVR training (cm)	22.0 (20.3–25.3)	27.0 (19.5–30.3)	0.65
BRS of lower limb before IVR training	V (V–VI)	VI (VI–VI)	0.13
FAC before IVR training	3 (2–3)	4 (4–4)	0.007 **
FES before IVR training (point)	29 (24–33)	34 (30–37)	0.17

Data are presented as mean ± standard deviation or median (interquartile range), as appropriate. Between-group comparisons were performed using the Mann–Whitney U test or Fisher’s exact test, as appropriate. The significance level was set at *p* < 0.05. *: *p* < 0.05, **: *p* < 0.01. Abbreviations: FIM: Functional Independence Measure; MMSE: Mini-Mental State Examination; BBS: Berg Balance Scale; FRT: Functional Reaching Test; BRS: Brunnstrom Recovery Stage; FAC: Functional Ambulation Categories; FES: Fall Efficacy Scale.

## Data Availability

The data that support the findings of this study are available from the corresponding author on request.

## Abbreviations

The following abbreviations are used in this manuscript:

QOL	Quality of life
VR	Virtual reality
IVR	Immersive virtual reality
HMD	Head-mounted display
BWS	Bodyweight supporting
BBS	Berg balance scale
FRT	Functional reaching test
10 MWT	10-m walk test
BRS	Brunnstrom recovery stage
FAC	Functional ambulation Categories
SSQ	Simulator sickness questionnaire
VAS	Visual analogue scale
FES	Fall efficacy scale
SD	Standard deviation
IQR	Interquartile range
FIM	Functional independent measure
MMSE	Mini-mental state examination
ROC	Receiver operating characteristic
AUC	Area under the curve
CI	Confidence interval
